# Association between steroid therapy and increased mortality in patients at risk for ARDS

**DOI:** 10.62838/jccm-2026-0026

**Published:** 2026-07-27

**Authors:** Sultan Almuntashiri, Andrew Bennett, Duo Zhang, Andrea Sikora, Aaron Chase

**Affiliations:** Department of Clinical Pharmacy, College of Pharmacy, University of Ha’il, Ha’il, Saudi Arabia; Department of Pharmacy, Augusta University Medical Center, Augusta, GA, USA; Department of Clinical and Administrative Pharmacy, College of Pharmacy, University of Georgia, Augusta, GA, USA; Department of Biomedical Informatics, School of Medicine, University of Colorado, Aurora, CO, USA

**Keywords:** ARDS, corticosteroids, dexamethasone, hydrocortisone

## Abstract

**Background:**

Corticosteroids are commonly used in critically ill patients with established risk factors for acute respiratory distress syndrome (ARDS), often for indications like sepsis or pneumonia, yet the choice of steroid and its impact on outcomes remain debated.

**Methods:**

We conducted a retrospective analysis of 160 ICU patients with documented clinical risk factors for ARDS at the time of ICU admission to evaluate the effect of corticosteroid therapy on hospital mortality. Clinical characteristics, treatment variables, and outcomes were compared between patients who received corticosteroids and those who did not. A subgroup exploratory analysis further compared outcomes between dexamethasone and hydrocortisone users. Logistic regression models were used to identify mortality predictors.

**Results:**

Of 160 patients, 91 (56.9%) received corticosteroids. Steroid-treated patients had higher Simplified Acute Physiology Score II (SAPS II) scores (54.4 vs. 48.0, p = 0.011), but no significant differences in age, partial pressure of arterial oxygen to fraction of inspired oxygen ratio (PaO_2_/FiO_2_), or mechanical ventilation use. Overall mortality was not significantly different between steroid and non-steroid users (42.9% vs. 33.3%, p = 0.221). Among steroid-treated patients, dexamethasone (n = 26) and hydrocortisone (n = 50) were the most frequently used agents. Mortality was significantly higher with hydrocortisone (58%) compared to dexamethasone (26.9%) (p = 0.010). In multivariate analysis, hydrocortisone use was associated with higher hospital mortality (adjusted OR = 4.41; 95% CI: 1.11–17.48; p = 0.035).

**Conclusion:**

Overall corticosteroid use was not associated with improved survival in patients with documented clinical risk factors for ARDS; however, in an exploratory analysis, hydrocortisone use was associated with higher hospital mortality than dexamethasone. Given the retrospective observational design and real-world factors influencing corticosteroid selection, including illness severity, these findings should be interpreted with caution and support the need for prospective studies to clarify these associations.

## Introduction

Acute respiratory distress syndrome (ARDS) is a severe and often fatal complication in critically ill patients, characterized by acute hypoxemia, diffuse pulmonary infiltrates, and non-cardiogenic pulmonary edema [1]. It commonly arises in the context of underlying conditions such as sepsis, pneumonia, trauma, or aspiration. Patients who are at risk of developing ARDS, those with systemic inflammation and acute respiratory compromise, represent a vulnerable population with high resource utilization and significant mortality [2]. In these patients, identifying factors associated with outcomes and treatment responses remains an ongoing challenge in critical care sitting.

The management of ARDS remains primarily supportive, relying on strategies such as lung-protective mechanical ventilation, conservative fluid management, and prone positioning to improve oxygenation and reduce ventilator-induced lung injury [3]. In addition to these supportive measures, several pharmacological therapies have been investigated to mitigate the underlying inflammatory processes associated with ARDS. These include corticosteroids, neuromuscular blocking agents (NMBAs), and beta-2 agonists, though none have shown consistent benefits across all patient populations [4]. In particular, corticosteroids have generated the most clinical interest due to their broad immunosuppressive and anti-inflammatory effects; however, their overall impact on outcomes—and the potential differences between specific agents—remain insufficiently defined in the existing literature [4].

Among the various corticosteroids used in ARDS, dexamethasone and hydrocortisone are the most commonly prescribed agents, yet they differ significantly in pharmacologic properties and clinical applications [5, 6]. Dexamethasone is a long-acting glucocorticoid with potent anti-inflammatory activity and no mineralocorticoid effect, making it attractive in the management of pulmonary inflammation. In contrast, hydrocortisone has both glucocorticoid and mineralocorticoid activity, which may support hemodynamic stability in septic patients but potentially contribute to fluid retention and worsen pulmonary edema in ARDS [4]. While dexamethasone is currently favored in guidelines, particularly following the randomized evaluation of COVID-19 therapy (RECOVERY) trial [7], hydrocortisone continues to be widely used in ICU settings—often for indications such as septic shock—without clear evidence of benefit in patients who subsequently develop ARDS [8].

In non-COVID ARDS, a large multicenter randomized controlled trial demonstrated that early administration of dexamethasone in patients with established moderate-to-severe ARDS significantly increased ventilator-free days and reduced 60-day mortality compared with standard care [5]. In contrast, a randomized controlled trial in patients with early sepsis-associated ARDS showed that hydrocortisone treatment improved pulmonary physiology and lung injury scores but was not associated with a significant survival benefit [6]. Taken together, these non-COVID ARDS studies suggest that while corticosteroids may improve respiratory physiology, clinical outcomes vary by agent and underlying disease context. In COVID-19–associated ARDS, dexamethasone has shown a consistent mortality benefit in large randomized trials, leading to its preferential recommendation, while other corticosteroids have demonstrated variable or neutral effects when used at equivalent doses [7, 9,10,11]. Overall, existing evidence highlights potential benefits of corticosteroids in ARDS but underscores substantial heterogeneity across patient populations, steroid agents, and treatment strategies, leaving uncertainty regarding optimal agent selection, particularly outside the COVID-19 setting.

Increasing evidence suggests that the timing of corticosteroid initiation is a critical determinant of treatment response in ARDS, with early administration showing more consistent benefits than delayed therapy in several experimental and clinical studies [12,13,14]. In contrast, once ARDS is fully established, prior trials have demonstrated heterogeneous effects of corticosteroids on mortality and other clinical outcomes [10, 11, 15]. Moreover, systemic corticosteroids are frequently initiated early in the course of illness for critical conditions such as sepsis, pneumonia, or shock—recognized clinical risk factors for ARDS—often before patients meet formal diagnostic criteria for established ARDS, such as persistent hypoxemia and radiographic evidence of acute lung injury [8, 16, 17]. In this early, high-risk setting, clinicians commonly choose between different corticosteroids based on clinical indication, institutional practice, or perceived safety, despite important pharmacologic differences between steroid agents [8, 18, 19]. Therefore, data directly comparing these corticosteroids in patients who are at early risk for ARDS remain limited, making it clinically relevant to explore whether early steroid selection in this phase is associated with differential outcomes. Given that hydrocortisone and dexamethasone differ substantially in pharmacologic profiles and are commonly selected for different early clinical indications, evaluating their association with outcomes in patients at risk for ARDS represents an underexplored area.

The objective of this study was to evaluate the association between corticosteroid use and hospital mortality in critically ill patients with documented clinical risk factors for ARDS, with an exploratory analysis comparing outcomes between hydrocortisone and dexamethasone. Given the retrospective observational design and real-world factors influencing corticosteroid selection, including illness severity and treatment indication, these findings warrant cautious interpretation.

## Material and Methods

### Study Design and Setting

This retrospective single-center cohort study was conducted at Augusta University Medical Center. Adult patients admitted to the ICU between September 2023 and March 2025 were screened for eligibility. The study was reviewed by the Augusta University Institutional Review Board and determined to be exempt. Due to the retrospective nature of the study and the use of de-identified clinical data, informed consent was waived. The study was conducted in accordance with the Declaration of Helsinki.

### Inclusion criteria

Patients were eligible if they were adults (≥ 18 years), admitted to the ICU during the study period and had at least one recognized clinical risk factor for ARDS documented at the time of ICU admission, including sepsis, pneumonia, aspiration, shock or other acute inflammatory conditions recorded in the electronic medical record.

### Exclusion criteria

Patients were excluded if they were younger than 18 years or had a documented diagnosis of ARDS recorded in the electronic medical record at the time of ICU admission.

### Corticosteroid exposure

Patients were categorized based on whether they received systemic corticosteroids during their ICU stay or not. Among patients who received systemic corticosteroids, the timing of corticosteroid initiation was recorded relative to ICU admission. Information on corticosteroid type was available for all treated patients; however, detailed data on equivalent dosing and treatment duration were not consistently documented and were therefore not included in the analysis. Corticosteroids were commonly initiated for clinical indications such as sepsis, pneumonia, or shock, consistent with routine ICU practice, although precise indications could not be uniformly ascertained retrospectively. During the study period, there was no standardized institutional protocol mandating corticosteroid use or specifying agent selection for patients at risk for ARDS. Treatment decisions were made at the discretion of the treating clinician.

### Data collection

Data were collected and reviewed from the institutional electronic medical records system. Key variables extracted included demographic characteristics, severity of illness scores, oxygenation status, corticosteroid type, and relevant clinical outcomes, including mechanical ventilation use, ICU length of stay, and hospital mortality.

### Outcomes

The primary outcome was hospital mortality. Secondary outcomes included ICU length of stay, hospital length of stay, and duration of mechanical ventilation.

### Statistical analysis

Categorical variables were analyzed using Chi-square, while continuous variables were analyzed using Student’s t-test or Mann–Whitney U test as appropriate. Logistic regression analyses were performed to evaluate the association between corticosteroid exposure and hospital mortality, with adjustment for key confounders. All statistical analyses were conducted using IBM SPSS Statistics version 30 (IBM Corp., Armonk, NY), and a p-value <0.05 was considered statistically significant.

## Result

Among 160 patients at risk for ARDS, 91 (56.9%) received corticosteroids. The median age was higher in the steroid group compared to non-steroid users (65 vs. 58 years, p = 0.073). Female distribution was similar between groups (44% vs. 50%, p = 0.450). The Simplified Acute Physiology Score II (SAPS II) score was significantly higher in the steroid group (54.4 ± 16.3 vs. 48.0 ± 15.0, p = 0.011), while Glasgow coma scale (GCS) scores were comparable (p = 0.925). The partial pressure of arterial oxygen to fraction of inspired oxygen ratio (PaO_2_/FiO_2_) ratio did not differ significantly between groups (p = 0.18). Mechanical ventilation was required in 33.8% of patients, with no difference between groups. The distribution of primary ARDS risk factors—such as aspiration, pneumonia, sepsis, and shock—did not differ significantly between the groups (p = 0.089). Mortality was numerically higher in the steroid group (42.9% vs. 33.3%, p = 0.221), but not statistically significant. Ventilator days, ICU length of stay, and hospital length of stay were similar across both groups. Patients who received corticosteroids had greater illness severity at baseline, reflected by significantly higher SAPS II scores and a higher prevalence of sepsis compared with non-steroid users (Table 1).

**Table 1.** Characteristics and clinical outcomes of patients at risk for ARDS

**Characteristic**	**Total cohort (n = 160)**	**Steroids (n = 91)**	**Non steroids (n = 69)**	**P-value**
Age	62.50 (43.25, 71.75)	65 (49, 72)	58 (39, 69)	0.073
Female sex, %	74 (46.5)	40 (44)	34 (50)	0.450
SAPS II score	51.66 (16) (95% CI 49.17–54.16)	54.43 (16.25) (95% CI 51.04–57.81)	48.01 (14.96) (95% CI 44.42–51.61)	0.011
GCS	15 (9, 15)	15 (9, 15)	15 (10.5, 15)	0.925
PaO_2_/FiO_2_	211 (136.16, 313.65)	220 (148, 316.66)	199 (101.5, 311.25)	0.18
Mechanical ventilation, n (%)	54 (33.8)	32 (35.2)	22 (31.9)	0.664
Primary ARDS risk factor				
Aspiration, n (%)	28 (17.5)	19 (20.9)	9 (13)	0.089
Pneumonia, n (%)	55 (34.4)	26 (28.6)	29 (42)	
Sepsis, n (%)	45 (28.1)	29 (31.9)	16 (23.2)	
Shock, n (%)	18 (11.3)	7 (7.7)	11 (15.9)	
Other, n (%)	14 (8.8)	10 (11)	4 (5.8)	
Patient outcomes				
Mortality, n (%)	62 (38.8) (95% CI 31.0–46.0)	39 (42.9) (95% CI 32.0–53.0)	23 (33.3) (95% CI 22.0–45.0)	0.221
Ventilator days	1.15 (0, 3.4)	1.08 (0, 3.36)	1.16 (0, 3.62)	0.528
ICU-LOS days	4.77 (2.05, 9.74)	4.40 (2.18, 11.06)	4.97 (1.90, 9.40)	0.737
Hospital LOS days	8.95 (3.80, 18)	8.08 (3.68, 16.87)	9.90 (3.82, 22.7)	0.521

Abbreviations: SAPS II: Simplified Acute Physiology Score II; GCS: Glasgow Coma Scale; PaO_2_: Partial pressure of oxygen; FiO_2_: fraction of inspired oxygen; LOS: Length of stay. Data are presented as mean ± standard deviation for normally distributed continuous variables, median (interquartile range) for non-normally distributed continuous variables, and number (percentage) for categorical variables. 95% confidence intervals are provided for key clinical outcomes and severity measures.

Next, we examined the types of corticosteroids administered among the treated group. Hydrocortisone was the most frequently used steroid (54.9%), followed by dexamethasone (28.6%) and methylprednisolone (8.8%). Less commonly used agents included prednisone (6.6%) and prednisolone (1.1%) (Table 2).

**Table 2.** Steroid types

**Steroid type**	**Number (n)**	**Percentage (%)**
Dexamethasone	26	28.6
Hydrocortisone	50	54.9
Methylprednisolone	8	8.8
Prednisolone	1	1.1
Prednisone	6	6.6
Total	91	100%

To further evaluate the association between steroid use and hospital mortality, logistic regression analyses were performed. In univariate analysis, steroid use was not significantly associated with mortality (OR = 1.50; 95% CI: 0.78–2.87; p = 0.222). After adjustment for major clinical confounders, steroid use remained non-significant (adjusted OR = 1.32; 95% CI: 0.65–2.70; p = 0.446). Among the covariates, only vasopressor use was independently associated with increased mortality (adjusted OR = 3.42; 95% CI: 1.396–8.398; p = 0.007) (Table 3).

**Table 3.** Logistic regression analyses for steroid use and mortality (all patients)

**Variable**	**P value**	**Odd ratio**	**95% CI Lower**	**95% CI Upper**
**Univariate analysis**				
Steroid use	0.222	1.500	0.783	2.874

**Multivariate analysis**				
Steroid use	0.446	1.320	0.646	2.697
Age	0.499	0.993	0.974	1.013
SAPS II	0.207	1.015	0.992	1.040
Mechanical ventilation	0.412	0.713	0.317	1.601
PaO_2_/FiO_2_	0.836	1.000	0.998	1.003
Sepsis	0.168	0.572	0.259	1.265
Shock	0.864	1.100	0.370	3.267
Vasopressor use	0.007	3.424	1.396	8.398

Abbreviations: SAPS II: Simplified Acute Physiology Score II; PaO_2_: Partial pressure of oxygen; FiO_2_: fraction of inspired oxygen.

Among patients who received corticosteroids, we further compared outcomes between dexamethasone and hydrocortisone users in a subgroup, exploratory analysis. Of the 76 patients analyzed, 26 received dexamethasone and 50 received hydrocortisone. Baseline characteristics were generally similar, though hydrocortisone users had a significantly higher SAPS II score (59.2 vs. 49.3, p = 0.006) and a higher proportion of female patients (52% vs. 26.9%, p = 0.036). There were no significant differences in age, GCS, PaO_2_/FiO_2_ ratio, mechanical ventilation, primary ARDS risk factor, ventilator days, ICU stay, or hospital length of stay between groups. However, hospital mortality was significantly higher in the hydrocortisone group (58%) compared to dexamethasone (26.9%) (p = 0.010) (Table 4).

**Table 4.** Characteristics and clinical outcomes of patients at risk for ARDS (patients received steroids)

**Characteristic**	**Total cohort (n = 76)**	**Dexamethasone (n = 26)**	**Hydrocortisone (n = 50)**	**P-value**
Age	64 (49, 72)	63.5 (36.75, 72)	64.5 (52.5, 72)	0.480
Female sex, n (%)	33 (43.4)	7 (26.9)	26 (52)	0.036
SAPS II score	55.83 (15.33) (95% CI 52.33–59.33)	49.27 (16.87) (95% CI 42.46–56.08)	59.24 (13.42) (95% CI 55.43–63.05)	0.006
GCS	15 (9, 15)	15 (8.75, 15)	15 (9, 15)	0.658
PaO_2_/FiO_2_	211.25 (138.25, 324.75)	208.75 (150.75, 414.25)	212.50 (137.66, 315.42)	0.361
Mechanical ventilation n (%)	26 (34.2)	10 (38.5)	16 (32)	0.573

Primary ARDS risk factor				
Aspiration, n (%)	14 (18.4)	2 (7.7)	12 (24)	0.291
Pneumonia, n (%)	22 (28.9)	11 (42.3)	11 (22)	
Sepsis, n (%)	25 (32.9)	8 (30.8)	17 (34)	
Shock, n (%)	6 (7.9)	2 (7.7)	4 (8)	
Other, n (%)	9 (11.8)	3 (11.50)	6 (12)	

Patient outcomes				
Mortality, n (%)	36 (47.4) (95% CI 36.0–59.0)	7 (26.9) (95% CI 9.0–45.0)	29 (58) (95% CI 44.0–72.0)	0.010
Ventilator days	1.3 (0, 3.4)	1.58 (0, 5.35)	1.26 (0, 3.23)	0.650
ICU-LOS days	3.72 (2.11, 9.78)	3.95 (1.98, 7.38)	3.72 (2.23, 12.21)	0.525
Hospital LOS days	7.8(3.25, 16.84)	7.43 (2.21, 16.36)	8.01 (3.80, 17.08)	0.443

Abbreviations: SAPS II: Simplified Acute Physiology Score II; GCS: Glasgow Coma Scale; PaO_2_: Partial pressure of oxygen; FiO_2_: fraction of inspired oxygen; LOS: Length of stay. Data are presented as mean ± standard deviation for normally distributed continuous variables, median (interquartile range) for non-normally distributed continuous variables, and number (percentage) for categorical variables. 95% confidence intervals are provided for key clinical outcomes and severity measures.

To explore the impact of hydrocortisone specifically on mortality, we performed logistic regression analyses among steroid-treated patients. In the univariate model, hydrocortisone use was significantly associated with increased mortality (OR = 3.75; 95% CI: 1.34–10.53; p = 0.012). This association remained significant after adjustment for major clinical confounders (adjusted OR = 4.41; 95% CI: 1.11–17.48; p = 0.035). Other variables were not independently associated with mortality in the adjusted model. These findings highlight a potentially harmful association between hydrocortisone use and mortality in this patient population (Table 5).

**Table 5.** Logistic regression analyses for hydrocortisone and mortality (patients received steroids)

**Variable**	**P value**	**Odd ratio**	**95% CI Lower**	**95% CI Upper**
**Univariate analysis**				
Hydrocortisone	0.012	3.748	1.335	10.527

**Multivariate analysis**				
Hydrocortisone	0.035	4.406	1.111	17.476
Age	0.307	0.983	0.952	1.015
SAPS II	0.282	1.022	0.983	1.062
Mechanical ventilation	0.617	1.369	0.400	4.690
PaO_2_/FiO_2_	0.395	1.002	0.998	1.006
Sepsis	0.516	0.693	0.229	2.098
Shock	0.243	4.214	0.376	47.246
Vasopressor use	0.895	1.124	0.200	6.314

Abbreviations: SAPS II: Simplified Acute Physiology Score II; PaO_2_: Partial pressure of oxygen; FiO_2_: fraction of inspired oxygen.

## Discussion

In this retrospective study of critically ill patients with documented clinical risk factors for ARDS at the time of ICU admission, we found that overall corticosteroid use was not significantly associated with hospital mortality. In an exploratory analysis of corticosteroid agents, hydrocortisone use was associated with a higher risk of death compared with dexamethasone after adjustment for measured confounders. These findings suggest that steroid selection, rather than corticosteroid exposure alone, may be relevant in this population. Given the retrospective, real-world nature of this study, in which corticosteroid selection is influenced by illness severity and clinical indication, the observed associations require careful interpretation and warrant further prospective investigation.

Several previous studies have examined the use of corticosteroids in ARDS, with varying results. A recent meta-analysis by Li et al. [20] found reduced mortality in randomized trials, especially when using low doses or agents like methylprednisolone and dexamethasone, but showed increased mortality in observational studies with high-dose use. Another meta-analysis by Chang et al. [21], which included only randomized controlled trials, stated that corticosteroids reduce 28-day mortality without increasing adverse events. These results highlight the inconsistent evidence and suggest that the effect of corticosteroids may depend on patient population, steroid type, and dosing strategy. Although our study evaluated patients with documented clinical risk factors for ARDS rather than established ARDS, our findings support this variability and suggest that outcomes may be influenced by the specific steroid agent used rather than corticosteroid exposure alone.

Dexamethasone has been evaluated in several studies for its role in ARDS. In a multicenter randomized trial by Villar et al. [5], dexamethasone significantly increased ventilator-free days and reduced 60-day mortality in patients with moderate-to-severe ARDS. A separate meta-analysis by Feng et al. [22] confirmed that dexamethasone reduces all-cause mortality and improves ventilator-free status at 28 days. In a retrospective study focused on COVID-19-related ARDS, high-dose dexamethasone was associated with improved oxygenation and reduced inflammation [23]. Together, these studies suggest that dexamethasone may offer clinical benefit in established ARDS populations, particularly when used in appropriate doses and patient populations.

Several trials have examined the role of hydrocortisone in ARDS, with mixed results. In a randomized controlled trial of patients with sepsis-associated ARDS, Tongyoo et al. found that hydrocortisone significantly improved oxygenation and lung injury scores, but did not confer a survival benefit compared to placebo [6]. Similarly, in a three-arm randomized trial comparing equivalent anti-inflammatory doses of corticosteroids in COVID-19-related ARDS, Taher et al. reported a trend toward better clinical outcomes with dexamethasone, while hydrocortisone was less effective, with no significant difference in mortality or ICU outcomes between groups [10]. The randomized, embedded, multifactorial adaptive platform trial for community-acquired pneumonia (REMAP-CAP) evaluated hydrocortisone in critically ill COVID-19 patients and found that both fixed-dose and shock-dependent regimens had a high probability of superiority in terms of organ support-free days, but the trial was stopped early, and no statistically definitive benefit could be concluded [24]. Collectively, these studies suggest that hydrocortisone may modestly improve pulmonary parameters, but the survival benefit remains uncertain.

Our findings add clinical context to the ongoing debate regarding corticosteroid choice in critically ill patients with ARDS risk factors. While previous randomized controlled trials have demonstrated a mortality benefit with dexamethasone in ARDS, and hydrocortisone has shown mixed or neutral effects on mortality, our study provides complementary real-world observational data examining these agents in a high-risk ICU population. In this cohort, dexamethasone use was associated with lower hospital mortality compared with hydrocortisone. After adjusting for confounders, including SAPS II score, mechanical ventilation, and oxygenation status, hydrocortisone use remained associated with higher hospital mortality. Taken together, these findings suggest that steroid selection may be associated with differential outcomes in critically ill patients with ARDS risk factors and underscore the need for further prospective studies to clarify comparative effects.

Although hydrocortisone is commonly used in the management of septic shock, its role in patients who subsequently develop ARDS may warrant caution. Sepsis-induced ARDS presents a unique pathophysiological context where pulmonary endothelial injury, capillary leak, and fluid accumulation already compromise gas exchange [25]. Given hydrocortisone’s mineralocorticoid activity, it has been hypothesized that its use could promote sodium and water retention, which may be undesirable in patients with established lung injury. This is particularly concerning in ARDS, where fluid management is a cornerstone of care. In contrast, dexamethasone—lacking mineralocorticoid activity and having more potent anti-inflammatory effects—may better modulate the dysregulated immune response without a clear impact on fluid balance. In our study, hydrocortisone use was associated with higher mortality, while dexamethasone appeared more favorable. These findings suggest that therapeutic strategies may need to be tailored to the evolving phase of critical illness, as treatments effective in early or primary etiologies of ARDS may not translate to benefit once ARDS has developed.

One strength of our study is the direct comparison between hydrocortisone and dexamethasone use in critically ill patients with documented clinical risk factors for ARDS. We adjusted for important clinical factors such as illness severity, need for mechanical ventilation and vasopressor use. In addition, we used logistic regression models to confirm the association between hydrocortisone use and hospital mortality. However, the study has several limitations that should be acknowledged. First, it was retrospective and observational, which means we cannot confirm a causal relationship. Treatment selection may also reflect confounding by indication, as hydrocortisone is often prescribed to septic shock patients, which may have influenced observed outcomes despite adjustment for illness severity. The number of patients, especially in the dexamethasone group, was relatively small, which may reduce the statistical power. Another major limitation is that the study cohort was identified based on recognized ARDS clinical risk factors rather than a standardized ARDS risk prediction score, which may limit direct comparability with studies that use validated risk instruments. Lastly, detailed information on corticosteroid dosing, duration of therapy, and precise timing of initiation relative to ICU admission was not consistently available and therefore could not be fully analyzed.

In this retrospective observational study of critically ill patients with documented clinical risk factors for ARDS, overall corticosteroid use was not associated with a significant difference in hospital mortality compared with non-users. In an exploratory subgroup analysis, hydrocortisone use was associated with higher hospital mortality when compared to dexamethasone. Because corticosteroid selection in clinical practice is influenced by illness severity and treatment indication, these associations should be interpreted with appropriate caution. Further prospective studies are needed to confirm these findings and to better define the role of corticosteroid selection.
